# Dentists’ refusal to manage patients with HIV, tuberculosis, HBV, HCV, epilepsy, and financial limitations in Damascus, Syria: a cross-sectional study

**DOI:** 10.1038/s41405-025-00341-9

**Published:** 2025-06-20

**Authors:** Muhammad Oweis Makieh, Muhammad Ibrahim Lababidi, Ramah Eimad Makieh, Mahmoud Abdul-Hak

**Affiliations:** 1 Damascus University, Damascus, Syria; 2 Pediatric Dentistry, Damascus University, Damascus, Syria; 3 Oral Medicine Department, Damascus University, Damascus, Syria

**Keywords:** Dental epidemiology, Occupational health

## Abstract

**Objective:**

This study investigates refusal rates of dentists in Damascus, Syria, to manage patients who disclose that they are carriers of tuberculosis, human immunodeficiency virus (HIV), hepatitis B and C (HBV/HCV), and patients with dental phobia, asthma, epilepsy, patients unable to afford dental care, and children. The aims are to identify to what extent dentists refuse patients who are diagnosed carriers of certain blood born viruses, require extra measures, take a lot of time, or do not pay.

**Methods:**

A cross-sectional study was conducted in Damascus by distributing paper and electronic questionnaires to dental clinics based on the administrative divisions of the city.

**Results:**

A total of 246 responses were collected. The average years of dental practice among respondents was 9.39 ± 9.8. Rates of refusal were as follows: children (*n *= 55, 22.4%), tuberculosis (*n *= 176, 71.5%), HIV (*n *= 192, 78.0%), HBV/HCV (*n *= 98, 39.8%), dental phobia (*n *= 58, 23.6%), asthma (*n *= 12, 4.9%), and epilepsy (*n *= 73, 29.7%). Acceptance of patients with tuberculosis, HIV, and HBV/HCV was positively associated with greater years of experience. Dentists who graduated outside of Syria were more likely to accept treating patients with HIV and HBV/HCV. A significant correlation was found between refusal rates for patients with tuberculosis, HIV and HBV/HCV.

**Conclusions:**

The proportion of dentists in Damascus refusing to treat patients who disclose that they are carriers of tuberculosis, HIV/AIDS, and HBV/HCV was notably high. Managing patients who cannot afford treatment often involved reducing fees. The findings provide valuable insights into the systemic challenges in healthcare delivery and propose possible improvements in managing vulnerable population in resource-constrained settings.

## Introduction

In 2021, the World Health Organization (WHO) estimated that the incidence rate of tuberculosis (TB) was 112 per 100,000 population in the WHO Eastern Mediterranean Region, accounting for about 8% of all tuberculosis cases worldwide [1]. TB spreads through the air, caused by Mycobacterium tuberculosis, a highly contagious bacterium. Consequently, overcrowding _whether at home or in the workplace_ is a significant factor in susceptibility to tuberculosis [2].

The prevalence of human immunodeficiency virus (HIV) in the Eastern Mediterranean Region is 0.1%. However, the region faces a critical situation with HIV infections, with new infection cases increasing by 47% and deaths rising by 57% compared to 2010 [3]. By the end of 2019, ~420,000 people were living with HIV in the region, but only one in four were receiving antiretroviral therapy [3].

In some countries, HIV patients seeking dental care may face many challenges. For instance, 47.2% of dentists in India reportedly refused to treat HIV-positive patients [4]. while in Saudi Arabia, 82% of dentists held negative attitudes towards HIV patients [5]. Additionally, among patients who are concerned about the risk of infection during dental treatment, 47.4% rank AIDS as their primary fear, followed by hepatitis B and C, at 15.5% [6].

Moreover, many infections with viral hepatitis B, C, and D are acquired in healthcare settings [7]. According to a study by Hatzakis et al., the prevalence of hepatitis B in several Arab countries ranges from 0.75% to 9.9%, while hepatitis C prevalence ranges from 0.2% to 14.7%, with these potentially higher among healthcare workers [8].

Statistics indicate that 80% of the population experiences some degree of dental fear [9]. This phobia may be related to the dentist, dental procedures, or dental tools and is defined as an exaggerated and baseless fear [10]. Specific procedures are often required to manage these cases, depending on the type of phobia, the patients’ age, and medical history, which may prompt clinicians to refer them to specialists [11, 12].

Asthma is the most common chronic respiratory disease, particularly prevalent among children and those seeking dental care [13, 14]. Approximately 300 Million people worldwide suffer from asthma, and available evidence suggests that about 8% of the population in the Middle East is affected by this condition [15].

Epilepsy is one of the most common neurological disorders in 20 out of the 23 countries in the Eastern Mediterranean Region, with an estimated 4.7 million people affected. Although epilepsy is a largely manageable disorder, and relatively inexpensive medications are available. However, between 60% and 98% of patients in developing countries remain untreated [16].

Consequently, the high prevalence rate of these conditions means that they are commonly encountered in dental clinics, necessitating specific management procedures. This may lead to various consequences and complications for both the healthcare system and community. Understanding the extent of acceptance of these cases, along with reasons for refusal, is essential for assessing the challenges these patients face when seeking dental care. Furthermore, identifying reasons for refusal can highlight areas for improvement in the healthcare system.

For these reasons, this research aimed to determine the extent of acceptance and the reported reasons for refusal by dental clinics in Damascus to treat patients who disclose they are carriers of tuberculosis, acquired immunodeficiency syndrome (HIV/AIDS), hepatitis B, hepatitis C, and conditions such as myocardial infarction, asthma, hypoglycemia, epilepsy, dental phobia and children patients. Additionally, this study explores how dentists manage patients unable to afford treatment under current economic conditions. The aims are to identify to what extent dentists refuse patients who are diagnosed carriers of certain blood born viruses, require extra measures, take a lot of time, or do not pay.

## Methods

### Study design and approach

A cross-sectional study was conducted in Damascus to investigate the acceptance and refusal rates of dental clinics when providing care to patients who disclose that they are carriers of tuberculosis, HIV/AIDS, hepatitis B and C, and patients with dental phobia, asthma, epilepsy, history of myocardial infarction, hypoglycemia, and pediatric patients. Additionally, the study aimed to examine the reported reasons for refusal.

By screening previous researches which studied the percentage of clinics’ refusal to provide dental care to specific conditions, we focused on conditions which had high prevalence rates in the region, in addition to medical conditions that attend the clinics and display various degrees of treatment difficulty [4, 6, 16].

Possible reasons for refusal were identified by referencing the studies of Azodo et al. and Dudina et al. [6, 17], which addressed similar conditions. Based on these insights, an Arabic-language questionnaire was designed. This study was conducted and reported in accordance with the STROBE guidelines for observational studies [18].

### Ethics approval and consent to participate

Ethical approval for this study was obtained from the Ethics Committee and the Board of Scientific Research at the Faculty of Dentistry, Damascus University, as well as from the Ministry of Higher Education and Research (SSRC-013-12062024). Informed written consent was obtained from all participants prior to their participation in the study. Participants were fully informed about the study’s purpose, procedures, and benefits, and they had the opportunity to ask questions before providing consent.

### Questionnaire validity and reliability

The questionnaire was specifically designed for this study in Arabic and was reviewed linguistically by two Arabic language professors. An English version is available as Supplementary File No. 1. A pilot study was initially conducted by distributing the questionnaire in paper form to 15 dentists working in private dental clinics. The dentists were asked to complete the questionnaire and discuss the questions face-to-face with the authors. Reliability testing was then performed using SPSS V27, yielding a Cronbach’s Alpha of 0.740, which indicates acceptable internal consistency [19]. Based on these result, the questionnaire was adopted for the study.

The questionnaire was distributed to dentists in two formats: directly in paper form or electronically via Google Forms.

The questionnaire begins with three initial questions regarding the respondent’s university, years of practical experience, and area of specialization. Possible reasons for refusing to treat the studied conditions included the following: need for special protective measures, need for enhanced sterilization procedures, distrust of sterilization products, fear of infection transmission to dental staff, concern for the clinic’s reputation, and lack of sufficient knowledge about the condition. Participants were allowed to select multiple reasons, and a blank line was provided for additional reasons.

Regarding the management of patients unable to afford dental treatment, five options were presented: declining to provide treatment, referring the patient to a dental school, reducing the fee, offering the least expensive treatment even if suboptimal, and a space to suggest other approaches.

### Sample size

According to the Damascus Dentists Syndicate, there were 4411 practicing dentists in Damascus as of February 2024. Slovin’s formula was used to calculate the sample size, and with a margin of error set at 6.2%, the required sample size was determined to be 246.

### Dental clinics selection criteria

Questionnaires were distributed to dental clinics across the administrative divisions of Damascus. Based on the health sectors map provided by the Ministry of Health, the city was divided into nine localities: Al-Zahera & Midan sector, the Old City sector, Al-Mazzeh sector, Al-Muhajirin sector, Dummar sector, Kafarsoseh sector, Barzeh sector, Kossor & Kassaa sector, and Malki & Abu Rumaneh sector [20].

Data were collected from March 16 to April 17, 2024. Questionnaires were provided to the dentist either in paper form or electronically, via a link sent to their phone numbers or through a QR code that directed them to the electronic questionnaire when scanned.

### Statistical analysis

Data were processed using IBM SPSS statistics, version 27. Descriptive statistics were conducted for all independent and outcome variables. Spearman’s correlation was used to examine the relationships between variables. A *p*-value of <0.05 was considered significant at a 95% confidence level.

## Results

A total of 265 questionnaires were distributed to dentists working in private clinics across Damascus. Of these, 19 questionnaires were declined, resulting in a rejection rate of 7.13%. Thus, statistical analyses were conducted on 246 completed questionnaires. Data were collected through two methods, 131 (53.2%) were paper questionnaires, and 115 (46.7%) were electronic.

Among 246 participating dentists, 97 were general practitioners, and 149 were specialists. Years of experience ranged from 0 to 47 years, with a mean of 9.39 ± 9.8 years. Most participants, 233 (94.7%) were graduates of Syrian universities, while 13 (5.2%) had graduated from universities abroad, (Table 1).

**Table 1:** Distribution of dentists by specialty and university of graduation.

Specialization	*N*	Percent
General practitioner	97	39.4
Cosmetic dentistry	13	5.3
Pediatric dentistry	22	8.9
Oral maxillofacial surgery	29	11.8
Fixable prosthodontics	9	3.7
Endodontics	20	8.1
Oral medicine	9	3.7
Orthodontics	23	9.3
Periodontics	18	7.3
Removable prosthodontics	5	2.0
Laser dentistry	1	0.4
Total	246	100.0
**University name**	***N***	**Percent**
Damascus University	173	70.3
Aleppo University	4	1.6
International University of Science and Technology	16	6.5
Syrian Private University	18	7.3
Al Andalus University	2	0.8
Arab International University	1	0.4
Al Baath University	8	3.3
Tishreen University	3	1.2
Hama University	5	2.0
Tartous University	2	0.8
University of Kalamoon	1	0.4
Total	233	94.7
Outside Universities	13	5.3
Total	246	100.0

**Fig. 1:**
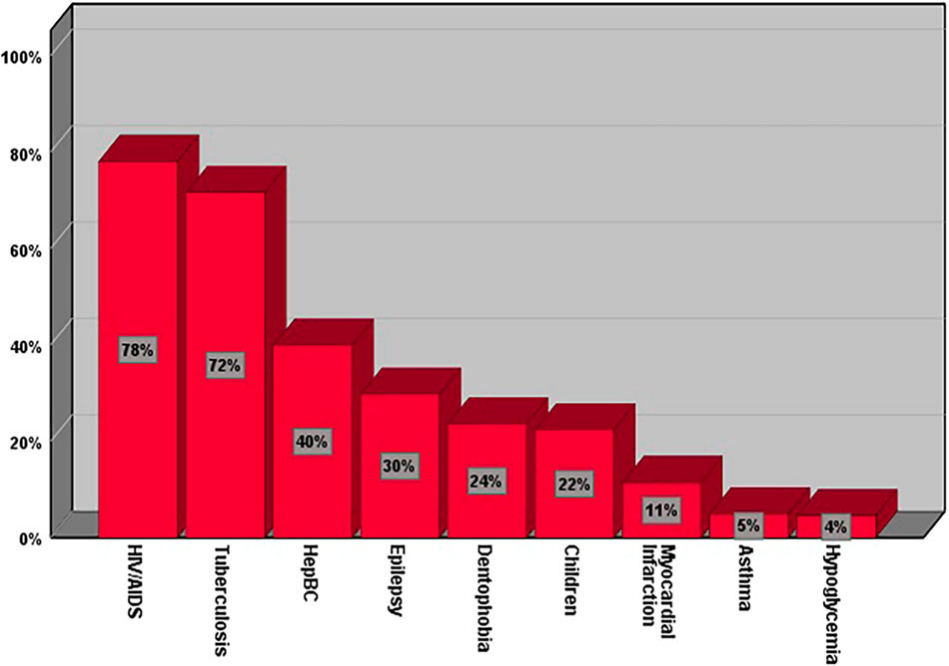
Percentages of dentists who refuse to provide care for each condition studied.

The percentages of dentists who refuse to provide dental care, by condition, are as follows in descending order: HIV/AIDS (*n *= 192, 78%), TB (*n *= 176, 71.5%), hepatitis B and C (*n *= 98, 39.8%), epilepsy (*n *= 73, 29.7%), dental phobia (*n *= 58, 23.6%), pediatric patient (*n *= 55, 22.4%), history of myocardial infarction (*n *= 28, 11.4%), asthma (*n *= 12, 4.9%), and hypoglycemia (*n *= 11, 4.5%), (Fig. 1).

Reasons for refusal of patients who disclose they are carriers of tuberculosis, HIV/AIDS, and hepatitis B and C, are presented in (Table 2). The primary reason for refusing patients with TB was fear of infection transmission to dental staff (29.0%). For HIV/AIDS patients, the most cited reason was the need for special protective procedures (32.4%), followed by the need for enhanced sterilization procedures (27.7%). Similarly, for patients with HBV/HCV the leading reason was the need for special protective procedures (36.8%), followed closely by the need for special sterilization procedures (34.5%).

**Table 2:** Reasons for refusal of patients with TB, HIV/AIDS, and HBV/HCV.

Refusal reasons	TB		HIV/AIDS		HBV/HCV	
	*N*	Percent	*N*	Percent	*N*	Percent
Need for special protection procedures	78	23.8	116	32.4	63	36.8
Need for special sterilization procedures	68	20.7	99	27.7	59	34.5
Distrust of sterilization products	31	9.5	48	13.4	23	13.5
Fear of infection transmission to staff	95	29.0	–	–	–	–
Fear for clinic reputation	7	2.1	15	4.2	10	5.8
Lack of sufficient knowledge about the condition	32	9.8	19	5.3	9	5.3
Ethical discomfort with AIDS patients	–	–	34	9.5	–	–
Other	17	5.2	27	7.5	7	4.1
Total	328	100.0	358	100.0	171	100.0

When asked about managing patients unable to afford dental treatment, nearly half of the dentists (45.0%) indicated they would reduce their fees, while (34.0%) would refer patients to a dental school, (Table 3).

**Table 3:** Reponses to managing patients unable to afford dental treatment.

Options	*N*	Percent
Apologizing and declining treatment	28	7.7
Referring to a dental school	123	34.0
Reducing fees	163	45.0
Offering minimal treatment even if inappropriate	21	5.8
Other	27	7.5
Total	362	100.0

Spearman’s correlation was applied to explore relationships between the studied variables. Significant statistical correlations are presented in (Table 4). Notably, a statistically significant correlation was found between refusal to treat patients with TB and refusal to treat those with HIV/AIDS and refusal to treat those with HBV/HCV (*P* < 0.05). Specifically, dentists who refused treatment for one of these conditions were more likely to refuse treatment for the others, while acceptance of one condition was associated with acceptance of the others.

**Table 4:** Significant correlations between variables studied.

		Lack of sufficient information about the condition		
		TB	HIV/AIDS	HBV/HCV
Electronic questionnaire	Correlation Coefficient	0.332**	0.165*	305**
	*P* value	*0.000*	*0.022*	*0.002*
	*N*	176	192	98
Damascus university/ Other universities	Correlation Coefficient	0.177*	0.108	0.199*
	*P* value	*0.018*	0.138	*0.049*
	*N*	176	192	98
		**Accepted treating / Marked yes**		
Syrian universities/Other universities		**TB**	**HIV/AIDS**	**HBV/HCV**
	Correlation Coefficient	−0.052	−0.138*	−0.155*
	*P* value	0.411	*.030*	*.015*
	*N*	246	246	246
Years of experience	Correlation Coefficient	−0.224**	−0.156*	−0.140*
	*P* value	*0.000*	*0.014*	*0.028*
	*N*	246	246	246

^*^Correlation is significant at the 0.05 level.

**Correlation is significant at the 0.01 level.

## Discussion

According to *Ethics of Medical Practice and its Legislation* by Dr. Hussein Noufal, published by Damascus University in 2020, “If you accept the patient, you must treat him well, and you have the right to refuse and not accept him if it is not an emergency case” [21]. This stance highlights the ethical obligation of healthcare providers to treat patients responsibly, yet it also supports their right to decline treatment in no-emergency cases. It is worth mentioning that there is no enforcement measures or penalties for refusing to provide dental care for either emergency or routine cases at the time of this study in Syria.

The FDI International Principles of Ethics for the Dental Profession notes the dentist’s right to refuse to provide dental care except for emergency care for humanitarian reasons. FDI also asserts that if dentists are not obligated to give any reason for refusing a patient, such condition may allow discrimination without the ability to hold them accountable. However, a dentist’s conscience may be the only way to prevent the abuse of human rights in this regard [22].

In Canada and the United States, current guidelines state that dentists must not refuse to treat a patient solely because they are infected with HIV or any other bloodborne disease [23, 24]. However, a study by McCarthy et al. in Canada found that one in six dentists (16.7%) reported refusing to treat patients infected with HIV. The reported reasons included a lack of belief in the ethical responsibility to treat such patients, and fear of transmission [25].

Consequently, in countries where guidelines obligate the treatment of all patients requesting care, dentists cannot refuse providing treatment based exclusively on a patient’s medical condition. However, they do have the right to refuse treatment for other reasons, such as the need for additional knowledge, skills, equipment or experience [26].

Healthcare workers in various positions are at risk of disease transmission during their clinical activities, as they are exposed to bloodborne infections by pathogens [27]. Patients infected with bloodborne pathogens, such as HIV, hepatitis B, and hepatitis C, may be difficult to diagnose through clinical examination or medical history. Therefore, OSHA’s (Occupational Safety and Health Administration) Bloodborne Pathogens Standard is based on Universal Precautions, which assume that all bodily fluids and sites are potentially infectious with pathogenic microorganisms [28]. In addition, Standard Precautions by the Centers for Disease Control and Prevention (CDC) are recommended for application in all patient care [29].

HBV, HCV and HIV are associated with significant morbidity and mortality rates; therefore, they are among the most concerning pathogens [28]. After a needlestick exposure to HIV-positive blood, the estimated risk of transmission is 0.3% per exposure. However, the risk of HBV transmission per exposure in dental offices can be as high as 30%. When exposed to HCV-contaminated blood, the risk of transmission is 1.8% [28]. As for Tuberculosis, transmission in dental settings is low. Despite this, the CDC recommends dental healthcare personnel implement protocols for tuberculosis infection control in dental offices [30]. Previous studies showed that HIV patients who receive antiretroviral therapy and have low levels of viral load and high CD4 cell counts had very low transmission rates to healthcare providers and therefore are safe to treat in dental clinics that apply universal precautions [31, 32]. However, it should also be noted that only one in four HIV infected individuals receive antiretroviral therapy in the Eastern Mediterranean region [3].

In this study, 78% of dentists in Damascus reported refusing to treat patients who reported to carry HIV/AIDS, a notably higher rate compared to the 46.3% refusal rate reported by El-Maaytah et al. [5] in Jordan, and the 5% refusal rate reported by Sears et al. [16] in the United States. In Nigeria, Utomi et al. [33] found that 78.4% of dentists indicate willingness to treat HIV infected individuals. In Taiwan, Hu et al. [34] found that about 50% of dental students were willing to treat patients with HIV, and more than 75% of them expressed willingness to treat HBV/HCV infected patients, compared to the 60% willingness rate for HBV/HCV infected patients found in this study. Several factors could account for these discrepancies, including cultural and community differences, variations in educational curricula, and legislation. Sallam et al. found that approximately two-thirds of Jordanian medical students displayed a positive attitude towards HIV/AIDS patients, while one-third displayed a negative attitude. [35]. During the Syrian war crisis, the study of Mohsen et al. found that (54.6%) of Syrian Private University’s medical students showed positive attitude towards HIV/HBV/HCV patients, while (45.4%) showed neutral attitude [36]. In our study, (*n *= 14, 77.8%) of dentists who graduated from Syrian Private University refused to treat HIV patients, and (*n *= 7, 38.9%) of those dentists refused to treat HBV/HCV patients. One can deduce that positive attitude does not necessarily imply acceptance of treating the patient at the clinic.

In this study, the most common reason for refusing to treat tuberculosis patients was the fear of infection transmission to dental staff, reported by 29% of participants. This concern is understandable due to the ease of aerosol transmission, which occurs more rapidly during coughing and to a lesser extent during speech and singing. Furthermore, larger respiratory droplets tend to fall to the ground, while smaller droplets may remain suspended in the air until they are either inhaled or ventilated out of the room [37].

The most frequently chosen option for assisting patients who cannot afford treatment was reducing fees, with 45% of participants opting for this approach. This indicates that offering discounted charges is a common practice among nearly half of the participants, likely in response to current economic conditions. This finding aligns with the observation that the second most popular option, chosen by 34% of participants, was referring patients to dental schools, where treatments are often provided free of charge or at nominal fees [14]. Additionally, some participants cited providing free dental treatment in emergency cases.

Significant correlations were found between the use of the electronic questionnaire and selecting “Lack of sufficient knowledge about the condition” as a reason for rejection for TB, HIV/AIDS, and HBV/HCV. Such correlations were not observed with the direct paper questionnaire. This may be due to the increased sense of privacy provided by the electronic questionnaire, which allows for greater anonymity compared to the paper format. This finding aligns with the observations of Kays et al. [38].

It is worth noting that there is no correlation between acceptance and rejection rates and the use of the electronic questionnaire for any of the conditions studied, indicating that the method of data collection does not influence the reported decision to accept or reject patients.

A significant positive correlation was found between years of experience and the acceptance of treating TB, HIV/AIDS, and HBV/HCV. This correlation may be attributed to several factors, including increased confidence among dentists in sterilization and protective methods, as well as enhanced ability to implement these methods effectively in their clinics with greater practical experience. Alternatively, it may be due to heightened awareness of the importance of treating these cases, thereby reducing the likelihood that patients will conceal their illnesses to receive necessary treatment. Previous studies on medical students had found that clinical students demonstrated a more positive attitude and higher levels of awareness compared to pre-clinical students, which may indicate a trend [35, 36].

During discussions with the authors while collecting data, participating dentists expressed concerns that patients might conceal their health conditions if dentists consistently refuse to treat them. This issue was discussed by Dudina et al., who found that concealing HIV/AIDS, is often linked to fears of confidentiality breaches, reduced access to quality healthcare, caregiver bias [17], and negative attitudes toward infected patients [39, 40].

A significant correlation was found between the acceptance of treating patients with HIV/AIDS and HBV/HCV among dentists who graduated from universities outside the country, with these dentists being more likely to accept patients with these conditions. This finding contrasts with the research by Sears et al., who found that dentists in Los Angeles who graduated from universities outside the United States were more likely to refuse treatment to HIV/AIDS patients [16]. it is possible that international graduates from outside Syria receive more appropriate training in precautionary measures for treating patients with these conditions, or they may be influenced by the regulations in their countries of education, which may mandate treatment and prohibit refusal of care. However, this observation does not apply to the acceptance of tuberculosis cases.

Interestingly, the rejection of one of these conditions - TB, HIV/AIDS, or HBV/HCV- is linked to the rejection of the others, which may suggest that some dentists employ a general aversion policy to treating potentially contagious conditions. Conversely, the willingness to provide dental treatment for one of these diseases is associated with the willingness to provide dental care to the others. To the best of our knowledge, no previous research has examined this correlation.

McCarthy et al. [25] and Oberoi et al. [4] found that (45% and 42.6% respectively) of dentists report that infection control procedures associated with HIV/AIDS patients place the dental clinic under financial strain. Seale et al. [41] found that 91% of general practitioner dentists in the US treat children, with only 9% refusing all children. The study of Doshi et al. [42] in India found that 8.8% of dentists noted that their clinic’s policy was to refuse to provide treatment for patients with epilepsy. Due to the comparatively high refusal rates in our study for non-infectious conditions such as children (*n *= 55, 22.4%) and epilepsy (*n *= 73, 29.7%), a business approach taken by refusing dentists should be taken into account, for example; these dentists may consider the extra time needed for treatment or the improved infection control procedures needed with certain conditions, which could influence the dentist’s decision. However, it should also be noted that the rates for dental care are not set by the government, which means the dentist can charge extra if needed, instead of outright refusal.

Hu et al. and Oberoi et al. found that better knowledge about HIV/HBV/HCV may be related to more willingness to treat infected individuals [4, 34].

A significant relationship was found between not graduating from Damascus University and selecting “lack of sufficient knowledge about the condition” as a reason for rejecting patients with tuberculosis and hepatitis B and C, but not for HIV/AIDS. This reason was more commonly chosen by those who did not graduate from Damascus University, suggesting that the curricula at Damascus University may provide superior education on tuberculosis and hepatitis B and C compared to other universities.

In the light of these findings, we recommend increasing attention to infection control courses in universities, with particular emphasis on methods for monitoring sterilization through regular examinations. University curricula should also be enriched with practical information on managing these diseases and education on the risks associated with refusing to treat such patients. Additionally, the role of the Dentists’ Syndicate could be strengthened through collaboration with the Infection Control Association to assess the level of infection control in private clinics and to enforce periodic inspections of sterilization equipment.

This study is limited in scope, focusing on a specific time and location, and is restricted to dentists practicing in private clinics within Damascus. This study groups HBV and HCV into one category, although HBV is vaccinated against while HCV is not vaccinated against in Syria, which might influence fear of infection transmission. Further research is needed to explore the underlying reasons and motivations for the high rejection rates identified in this study. Although this research was conducted in Damascus, similar studies in other Syrian cities would provide a broader understanding of this issue.

## Conclusions

The percentages of dentists refusing to treat patients who disclose they carry tuberculosis, HIV/AIDS, and hepatitis B and C were notably high in Damascus, at 78.0%, 71.5%, and 39.8%, respectively. An increase in years of experience was correlated with a higher acceptance rate for treating these conditions. Dentists commonly addressed the issue of patients unable to afford treatment by reducing fees. Additionally, there was a significant correlation between graduating from universities outside Syria and the acceptance of treating patients with HIV/AIDS and hepatitis B and C. This finding highlights the need to enhance Syrian university curricula and further strengthen the role of the Dentists’ Syndicate in continuing education. These findings offer suggestions for improving the management of healthcare for vulnerable patients in resource-limited settings.

## Supplementary information

Supplementary Information

## Data Availability

The datasets generated and analyzed during this study are not publicly available but can be obtained from the corresponding author upon reasonable request.

## References

[1] World Health organization. WHO EMRO, Epidemiological situation, Tuberculosis. WHO. 2024. Available from: https://www.emro.who.int/tuberculosis/epidemiological-situation/index.html.

[2] Chong XM, McClean L, McMaster P. Tuberculosis: implications for dentistry. Dent Update. 2024;51:258–62.

[3] World Health Organization. WHO EMRO, World AIDS campaigns 2020. WHO. 2024. Available from: https://www.emro.who.int/ar/world-aidscampaigns/wad2020/index.html.

[4] Oberoi SS, Kapoor S, Rekhi A, Sachdeva S. A cross-sectional study to assess the knowledge and attitude of the private dental practitioners towards the treatment of HIV/AIDS-infected Individuals. West Indian Med J. 2022;69:559–64.

[5] El-Maaytah M, Al Kayed A, Al Qudah M, Al Ahmad H, Al-Dabbagh W, Jerjes K, et al. Willingness of dentists in Jordan to treat HIV-infected patients. Oral Dis. 2005;11:318–22. 16120120 10.1111/j.1601-0825.2005.01126.x

[6] Azodo CC, Umoh A, Oboro HO, Ehizele AO, Ezeja EB. Expectations and perceptions of Nigerian patients regarding infectious diseases in dentistry. Odontostomatol Trop. 2011;34:27–32. 21682216

[7] De Pascalis CoppolaN, Onorato S, Calò L, Sagnelli F, Sagnelli C. E. Hepatitis B virus and hepatitis C virus infection in healthcare workers. World J Hepatol. 2016;8:273. 26925201 10.4254/wjh.v8.i5.273 PMC4757650

[8] Hatzakis A, Van Damme P, Alcorn K, Gore C, Benazzouz M, Berkane S, et al. The state of hepatitis B and C in the Mediterranean and Balkan countries: report from a summit conference. J Viral Hepat. 2013;20:1–20. 23827008 10.1111/jvh.12120

[9] Aburas S, Pfaffeneder-Mantai F, Hofmann A, Meller O, Schneider B, Turhani D. Dentophobia and dental treatment: an umbrella review of the published literature. Spec Care Dent. 2023;43:163–73. 10.1111/scd.12749 35700448

[10] Robo I. Dental phobia, summary of information published on this term. Biomed J Sci Tech Res. 2020;30:23631–4.

[11] Klingberg G, Broberg AG. Dental fear/anxiety and dental behaviour management problems in children and adolescents: a review of prevalence and concomitant psychological factors. Int J Paediatr Dent. 2007;17:391–406. 17935593 10.1111/j.1365-263X.2007.00872.x

[12] Abdalhai R, Kouchaji C, Alkhatib R. The effect of aromatherapy with Lavender-Neroli oil and music in management of pediatric dental anxiety: a randomized control trial. BDJ open. 2024;10:5. 38286818 10.1038/s41405-024-00186-8 PMC10825141

[13] Ferrante G, La Grutta S. The burden of pediatric asthma. Front Pediatr. 2018;6:186. 29988370 10.3389/fped.2018.00186 PMC6023992

[14] Almonaqel MB, Makieh RE. Health status and visit reasons for children attending the Pediatric Dentistry department in damascus university, damascus, Syria: a retrospective study. Saudi Dent J. 2024;36:1025–30. 39035554 10.1016/j.sdentj.2024.05.009 PMC11255921

[15] World Health Organization. Asthma. Health topics - WHO EMRO. WHO. 2024. Available from: https://www.emro.who.int/health-topics/asthma/index.html.

[16] Sears B, Cooper C, Younai FS, Donohoe T. HIV discrimination in dental care: results of a testing study in Los Angeles County. Loyola Los Angel Law Rev. 2011;45:909–62. Available from: http://heinonlinebackup.com/hol-cgi-bin/get_pdf.cgi?handle=hein.journals/lla45&section=34.

[17] Dudina VI, King EJ, Tsareva AV. Concealing an HIV-positive status in medical settings: discussions in russian online forums. Qual Health Res. 2020;30:1379–91. 32558635 10.1177/1049732320914574

[18] Von Elm E, Altman DG, Egger M, Pocock SJ, Gøtzsche PC, Vandenbroucke JP. The strengthening the reporting of observational studies in epidemiology (STROBE) statement: guidelines for reporting observational studies. Lancet. 2007;370:1453–7. 18064739 10.1016/S0140-6736(07)61602-X

[19] Ranganathan P, Caduff C, Frampton CMA. Designing and validating a research questionnaire - Part 2. Perspect Clin Res. 2024;15:42–5. 38282630 10.4103/picr.picr_318_23 PMC10810057

[20] Ballouk MAH, Dashash M. Caries prevalence and dental health of 8-12 year-old children in Damascus city in Syria during the Syrian Crisis; a cross-sectional epidemiological oral health survey. BMC Oral Health. 2019;19:1–6. 30646889 10.1186/s12903-019-0713-9 PMC6332908

[21] Hussein N. Ethics of medical practice and its legislation. Damascus: Damascus University; 2020;158.

[22] Williams JR. Dental ethics manual. First edit. FDI World Dental Federation; 2007. Available from: https://www.fdiworlddental.org/resource/dental-ethics-manual.

[23] Association CD. Statement on the ethical and legal consideration of treating patients with infectious diseases. J Can Dent Assoc. 1988;54:385. 10518342

[24] on Dental ADAC. Facts about AIDS for the Dental Team. J Am Dent Assoc. 1988;117:i–iii.

[25] McCarthy GM, Koval JJ, MacDonald JK. Factors associated with refusal to treat HIV-infected patients: the results of a national survey of dentists in Canada. Am J Public Health. 1999;89:541–5. 10191798 10.2105/ajph.89.4.541 PMC1508900

[26] Graeber JJ. Am I obligated to treat a patient who is HIV-positive?. J Am Dent Assoc. 2004;135:1172–3.

[27] Gerberding JL. Incidence and prevalence of human immunodeficiency virus, Hepatitis B Virus, Hepatitis C Virus, and cytomegalovirus among health care personnel at risk for blood exposure: final report from a longitudinal study. J Infect Dis. 1994;170:1410–7. 7995979 10.1093/infdis/170.6.1410

[28] Denault D, Gardner H. OSHA bloodborne pathogen standards. StatPearls. 2025. 34033323

[29] Sebastiani FR, Dym H, Kirpalani T. Infection control in the dental office. Dent Clin North Am. 2017;61:435–57. 28317575 10.1016/j.cden.2016.12.008

[30] Research Services and Scientific Information AL& A. Tuberculosis. American Dental Association. 2023. Available from: https://www.ada.org/resources/ada-library/oral-health-topics/tuberculosis-overview-and-dental-treatment-conside.

[31] Montaner JS, Lima VD, Barrios R, Yip B, Wood E, Kerr T, et al. Association of highly active antiretroviral therapy coverage, population viral load, and yearly new HIV diagnoses in British Columbia, Canada: a population-based study. Lancet. 2010;376:532–9. 20638713 10.1016/S0140-6736(10)60936-1 PMC2996043

[32] Novitsky V, Essex M. Using HIV viral load to guide treatment-for-prevention interventions. Curr Opin HIV AIDS. 2012;7:117–24. 22258501 10.1097/COH.0b013e32834fe8ff

[33] Utomi IL, Onajole AT, Arotiba JT. HIV/AIDS: knowledge and attitudes of dentists in South-Western Nigeria. Niger J Heal Biomed Sci. 2008;7. Available from: http://www.ajol.info/index.php/njhbs/article/view/11661.

[34] Hu SW, Lai HR, Liao PH. Comparing dental students’ knowledge of and attitudes toward Hepatitis B Virus-, Hepatitis C Virus-, and HIV-infected patients in Taiwan. AIDS Patient Care STDS. 2004;18:587–93. 15630786 10.1089/apc.2004.18.587

[35] Sallam M, Alabbadi AM, Abdel-Razeq S, Battah K, Malkawi L, Al-Abbadi MA, et al. HIV knowledge and stigmatizing attitude towards people living with HIV/AIDS among medical students in Jordan. Int J Environ Res Public Health. 2022;19:745. 35055566 10.3390/ijerph19020745 PMC8775845

[36] Mohsen F, Shibani M, Ibrahim N, Alhourani G, Melhem S, Alzabibi MA, et al. Knowledge, attitude, and practice regarding HIV, HBV, and HCV among medical students of Syrian Private University, Damascus, Syria. Community Heal Equity Res Policy. 2023;43:161–70. 10.1177/0272684X211004923 33823689

[37] Yates TA, Khan PY, Knight GM, Taylor JG, McHugh TD, Lipman M, et al. The transmission of Mycobacterium tuberculosis in high burden settings. Lancet Infect Dis. 2016;16:227–38. 26867464 10.1016/S1473-3099(15)00499-5

[38] Kays K, Gathercoal K, Buhrow W. Does survey format influence self-disclosure on sensitive question items?. Comput Hum Behav. 2012;28:251–6.

[39] Alshouibi E, Alaqil F. HIV-related discrimination among senior dental students in Jeddah. J Int Soc Prev Community Dent. 2019;9:219–24. 31198692 10.4103/jispcd.JISPCD_420_18 PMC6559049

[40] Arheiam A, El Tantawi M, Al-Ansari A, Ingafou M, El Howati A, Gaballah K, et al. Arab dentists’ refusal to treat HIV positive patients: a survey of recently graduated dentists from three Arab dental schools. Acta Odontol Scand. 2017;75:355–60. 28431481 10.1080/00016357.2017.1316867

[41] Seale NS, Casamassimo PS. Access to dental care for children in the United States. J Am Dent Assoc. 2003;134:1630–40. 14719761 10.14219/jada.archive.2003.0110

[42] Doshi D, Reddy BS, Kulkarni S, Karunakar, P NA. Dentists’ knowledge, attitudes and practices toward patients with epilepsy in Hyderabad city, India. Epilepsy Behav. 2012;23:447–50. 22381393 10.1016/j.yebeh.2012.01.022

