# Supplementary material for: Dentists’ refusal to manage patients with HIV, tuberculosis, HBV, HCV, epilepsy, and financial limitations in Damascus, Syria: a cross-sectional study
Source: BDJ Open. 2025 Jun 20;11:60. doi: 10.1038/s41405-025-00341-9 (PMC12181348; doi:10.1038/s41405-025-00341-9)
Supplement: Supplementary file 1 — Supplementary Information [file 41405_2025_341_MOESM1_ESM.pdf]

Research Participant Form

- Number of years in dental practice… ....…………………………………………...
- University graduated from… ....…………………………..……………..
- Specialization… ....……………………………………....…………..

---

1. Do you offer dental treatment for tuberculosis patients in this clinic?

☐ Yes

☐ No

<u>If no, please indicate reasons (select all that apply):</u>

☐ The need for special protective procedures.
☐ The need for specialized sanitization procedures.
☐ Distrust in sanitization products.
☐ Fear of infection transmission to dental staff.
☐ Concern for the clinic's reputation.
☐ Lack of sufficient information about this disease.
☐ Other: … …… …….

2. Do you offer dental treatment for people living with HIV in this clinic?

☐ Yes

☐ No

<u>If no, please indicate reasons (select all that apply):</u>

☐ The need for special protective procedures.
☐ The need for specialized sanitization procedures.
☐ Distrust in sanitization products.
☐ Concern for the clinic's reputation.
☐ Ethical concerns when treating these patients.
☐ Lack of sufficient information about this disease.
☐ Other: … …… …….

3. Do you offer dental treatment for people with hepatitis B and C (HBV/HCV) in this clinic?

☐ Yes

☐ No

<u>If no, please indicate reasons (select all that apply):</u>

☐ The need for special protective procedures.
☐ The need for specialized sanitization procedures.
☐ Distrust in sanitization products.
☐ Concern for the clinic's reputation.
☐ Lack of sufficient information about this disease.
☐ Other: … …… …….

4. **How do you handle cases where patients cannot afford dental treatment?** (Multiple selections allowed)

☐ Refuse to provide dental care.
☐ Refer to a dental school.
☐ Reduce the fees.
☐ Provide a lower-cost treatment, even if it is not the ideal procedure.
☐ Other: … …… …….

5. **Do you provide dental treatment for patients with the following conditions:** (Select Yes or No for each)

A. Pediatric patients.	☐ Yes	☐ No
B. Dentophobia (fear of dental treatment).	☐ Yes	☐ No
C. History of heart attack.	☐ Yes	☐ No
D. Asthma.	☐ Yes	☐ No
E. Hypoglycemia.	☐ Yes	☐ No
F. Epilepsy.	☐ Yes	☐ No
